# 2-(Ethyl­sulfin­yl)imidazo[1,2-*a*]pyridine-3-sulfonamide

**DOI:** 10.1107/S1600536812013992

**Published:** 2012-04-13

**Authors:** Yaling Gong, Haixia Ma, Jing Li

**Affiliations:** a Institute of Materia Medica, Chinese Academy of Medical Sciences & Peking Union Medical College, Beijing 100050, People’s Republic of China; b School of Chemistry and Chemical Engineering, Shanxi University, Taiyuan 030006, People’s Republic of China

## Abstract

The supra­molecular structure of the title compound, C_9_H_11_N_3_O_3_S_2_, is defined by two inter­molecular hydrogen bonds. Pairs of N—H⋯N hydrogen bonds link the mol­ecules into centrosymmetric dimers and N—H⋯O hydrogen bonds link the dimers into a tubular chain structure running parallel to the *a* axis.

## Related literature

The title compound is a derivative of sulfosulfuron [systematic name: 1-(4,6-dimeth­oxy­pyrimidin-2-yl)-3-(2-ethyl­sulfonyl­imid­azo[1,2-*a*]pyridin-3-ylsulfon­yl)urea], a high-performance sulfonyl­urea herbicide used to control several grassy weeds in wheat, see: Maxwell *et al.* (2005 ▶).
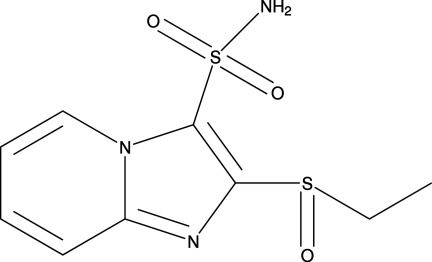


## Experimental

### 

#### Crystal data

C_9_H_11_N_3_O_3_S_2_
*M* *_r_* = 273.33
Triclinic, 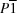

*a* = 8.3761 (9) Å
*b* = 8.5438 (9) Å
*c* = 9.1083 (10) Å
α = 88.832 (2)°
β = 75.376 (1)°
γ = 65.170 (1)°
*V* = 569.67 (11) Å^3^
*Z* = 2
Mo *K*α radiation
μ = 0.47 mm^−1^
*T* = 296 K
0.30 × 0.20 × 0.20 mm

#### Data collection

Bruker SMART APEX CCD diffractometer
Absorption correction: multi-scan (*SADABS*; Sheldrick, 1996 ▶) *T*_min_ = 0.873, *T*_max_ = 0.912
6015 measured reflections
2001 independent reflections
1793 reflections with *I* > 2σ(*I*)
*R*_int_ = 0.017

#### Refinement

*R*[*F* ^2^ > 2σ(*F* ^2^)] = 0.026
*wR*(*F* ^2^) = 0.068
*S* = 1.06
2001 reflections
155 parameters
H-atom parameters constrained
Δρ_max_ = 0.29 e Å^−3^
Δρ_min_ = −0.30 e Å^−3^

### 

Data collection: *SMART* (Siemens, 1996 ▶); cell refinement: *SAINT* (Siemens, 1996 ▶); data reduction: *SAINT*; program(s) used to solve structure: *SHELXS97* (Sheldrick, 2008 ▶); program(s) used to refine structure: *SHELXL97* (Sheldrick, 2008 ▶); molecular graphics: *PLATON* (Spek, 2009 ▶); software used to prepare material for publication: *SHELXL97*.

## Supplementary Material

Crystal structure: contains datablock(s) I, global. DOI: 10.1107/S1600536812013992/go2050sup1.cif

Structure factors: contains datablock(s) I. DOI: 10.1107/S1600536812013992/go2050Isup2.hkl

Supplementary material file. DOI: 10.1107/S1600536812013992/go2050Isup3.cml

Additional supplementary materials:  crystallographic information; 3D view; checkCIF report

## Figures and Tables

**Table 1:** Hydrogen-bond geometry (Å, °)

*D*—H⋯*A*	*D*—H	H⋯*A*	*D*⋯*A*	*D*—H⋯*A*
N3—H3*A*⋯O1^i^	0.83	2.07	2.888 (2)	171
N3—H3*B*⋯N1^ii^	0.82	2.24	3.026 (2)	161

Symmetry codes: (i) 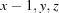; (ii) 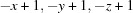.

**Fig. 1.**
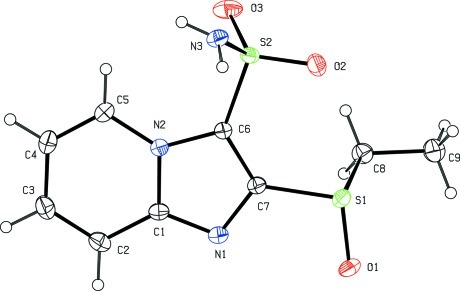
A view of (1) with our numbering scheme. Displacement ellipsoids are drawn at the 30% probability level.

**Fig. 2.**
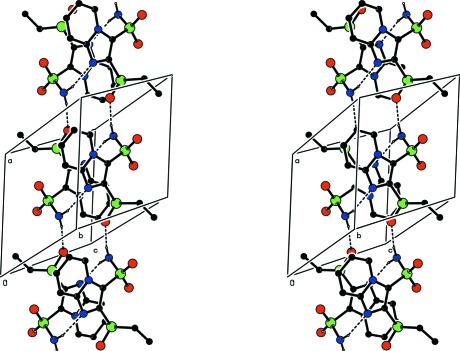
A stereoview of part of the crystal structure of compound, showing the tubular chain structure which runs parallel to the a-axis. Hydrogen atoms not involved in the motifs are not included.

## supplementary crystallographic information

### Comment

Sulfosulfuron,1-(4,6-dimethoxypyrimidin-2-yl)-3-(2-ethylsulfonylimidazo [1,2-a]pyridin-3-ylsulfonyl)urea, is high-performance sulfonylurea herbicide, and can effectively control several grassy weeds in wheat (Maxwell, *et al.*2005). In the course of exploring its derivatives, we obtained the compound C_9_H_11_N_3_O_3_S_2_, Figure, 1.

The supramolecular structure is defined by the N3—H3B···N1 hydrogen bond which links the molecules into centrosymmetric dimers lying across the centre-of-symmetry at (0.5,0.5,0.5) and the N3–H3B···O1 hydrogen bond which links the dimers into tubular chains which run parallel to the *a*-axis, Table 1 and Figure 2.

### Experimental

*m*-chloroperoxybenzoic acid (1.88 g, 8.22 mmol) in 100 ml CH_2_Cl_2_ was added dropwise to a solution of 2-ethylthioimidazo[1,2-a]pyridine-3-sulfonamide (2.2 g, 8.22 mmol) in 200 ml CH_2_Cl_2_ in an ice water bath. The suspension was stirred at 0–5°C for more than 3 h, and filtered. After removing the solvent, and the crude product was recrystallized in MeOH to give white crystalline product (1.24 g, 55% yield)). The melting point of the product was 203–205°C.

### Refinement

H atoms were treated as riding atoms with C—H(aromatic), 0.93 Å, and C—H(CH_2_), 0.97\$A with *U*_iso_ = 1.2Ueq(C) and *C*—*H*(methyl), 0.96 Å, with *U*_iso_ =1.5Ueq(C).

The hydrogen atoms attached to N3 were located on a difference Fourier map and allowed to ride at these positions. These positions were confirmed in a final difference Fourier map.

### Crystal data

**Table:**

C_9_H_11_N_3_O_3_S_2_	*Z* = 2
*M**_r_* = 273.33	*F*(000) = 284
Triclinic, *P*1	*D*_x_ = 1.593 Mg m^−^^3^
Hall symbol: -P 1	Mo *K*α radiation, λ = 0.71073 Å
*a* = 8.3761 (9) Å	Cell parameters from 3732 reflections
*b* = 8.5438 (9) Å	θ = 2.6–30.8°
*c* = 9.1083 (10) Å	µ = 0.47 mm^−^^1^
α = 88.832 (2)°	*T* = 296 K
β = 75.376 (1)°	Block, colourless
γ = 65.170 (1)°	0.30 × 0.20 × 0.20 mm
*V* = 569.67 (11) Å^3^	

### Data collection

**Table:**

Bruker SMART APEX CCD diffractometer	2001 independent reflections
Radiation source: fine-focus sealed tube	1793 reflections with *I* > 2σ(*I*)
Graphite monochromator	*R*_int_ = 0.017
phi and ω scans	θ_max_ = 25.0°, θ_min_ = 2.6°
Absorption correction: multi-scan (*SADABS*; Sheldrick, 1996)	*h* = −9→9
*T*_min_ = 0.873, *T*_max_ = 0.912	*k* = −10→10
6015 measured reflections	*l* = −10→10

### Refinement

**Table:**

Refinement on *F*^2^	Primary atom site location: structure-invariant direct methods
Least-squares matrix: full	Secondary atom site location: difference Fourier map
*R*[*F*^2^ > 2σ(*F*^2^)] = 0.026	Hydrogen site location: inferred from neighbouring sites
*wR*(*F*^2^) = 0.068	H-atom parameters constrained
*S* = 1.06	*w* = 1/[σ^2^(*F*_o_^2^) + (0.0298*P*)^2^ + 0.3093*P*] where *P* = (*F*_o_^2^ + 2*F*_c_^2^)/3
2001 reflections	(Δ/σ)_max_ = 0.001
155 parameters	Δρ_max_ = 0.29 e Å^−^^3^
0 restraints	Δρ_min_ = −0.30 e Å^−^^3^

### Special details

**Table:**

Geometry. All esds (except the esd in the dihedral angle between two l.s. planes) are estimated using the full covariance matrix. The cell esds are taken into account individually in the estimation of esds in distances, angles and torsion angles; correlations between esds in cell parameters are only used when they are defined by crystal symmetry. An approximate (isotropic) treatment of cell esds is used for estimating esds involving l.s. planes.
Refinement. Refinement of *F*^2^ against ALL reflections. The weighted *R*-factor *wR* and goodness of fit *S* are based on *F*^2^, conventional *R*-factors *R* are based on *F*, with *F* set to zero for negative *F*^2^. The threshold expression of *F*^2^ > σ(*F*^2^) is used only for calculating *R*-factors(gt) *etc*. and is not relevant to the choice of reflections for refinement. *R*-factors based on *F*^2^ are statistically about twice as large as those based on *F*, and *R*- factors based on ALL data will be even larger.

### Fractional atomic coordinates and isotropic or equivalent isotropic displacement parameters (Å^2^)

**Table:**

	*x*	*y*	*z*	*U*_iso_*/*U*_eq_	
S1	0.85430 (6)	0.27576 (6)	0.34364 (5)	0.02656 (13)	
S2	0.45246 (6)	0.23968 (6)	0.29742 (5)	0.02731 (13)	
O1	0.96772 (17)	0.30562 (18)	0.43380 (16)	0.0388 (3)	
O2	0.59485 (18)	0.2537 (2)	0.18084 (15)	0.0431 (4)	
O3	0.4246 (2)	0.08604 (17)	0.29993 (17)	0.0435 (4)	
N1	0.64659 (19)	0.30840 (18)	0.63394 (16)	0.0267 (3)	
N2	0.39592 (19)	0.28563 (17)	0.61067 (15)	0.0235 (3)	
N3	0.2631 (2)	0.39803 (19)	0.29715 (17)	0.0303 (3)	
H3A	0.1735	0.3815	0.3436	0.036*	
H3B	0.2634	0.4922	0.3130	0.036*	
C1	0.4861 (2)	0.3112 (2)	0.7099 (2)	0.0255 (4)	
C2	0.4020 (3)	0.3362 (2)	0.8674 (2)	0.0335 (4)	
H2B	0.4593	0.3540	0.9364	0.040*	
C3	0.2362 (3)	0.3342 (3)	0.9171 (2)	0.0373 (4)	
H3D	0.1791	0.3512	1.0210	0.045*	
C4	0.1494 (3)	0.3065 (3)	0.8128 (2)	0.0355 (4)	
H4A	0.0354	0.3060	0.8490	0.043*	
C5	0.2291 (2)	0.2807 (2)	0.6613 (2)	0.0292 (4)	
H5A	0.1726	0.2601	0.5930	0.035*	
C6	0.5075 (2)	0.2671 (2)	0.46454 (19)	0.0240 (4)	
C7	0.6586 (2)	0.2806 (2)	0.48511 (19)	0.0241 (4)	
C8	0.9606 (2)	0.0468 (2)	0.2813 (2)	0.0322 (4)	
H8A	0.9966	−0.0194	0.3646	0.039*	
H8B	0.8738	0.0151	0.2517	0.039*	
C9	1.1260 (3)	0.0039 (3)	0.1481 (2)	0.0439 (5)	
H9A	1.1791	−0.1173	0.1144	0.066*	
H9B	1.2138	0.0309	0.1787	0.066*	
H9C	1.0904	0.0707	0.0663	0.066*	

### Atomic displacement parameters (Å^2^)

**Table:**

	*U*^11^	*U*^22^	*U*^33^	*U*^12^	*U*^13^	*U*^23^
S1	0.0231 (2)	0.0276 (2)	0.0309 (2)	−0.01298 (18)	−0.00662 (17)	0.00240 (17)
S2	0.0250 (2)	0.0318 (2)	0.0257 (2)	−0.01139 (19)	−0.00860 (17)	−0.00373 (17)
O1	0.0293 (7)	0.0504 (8)	0.0445 (8)	−0.0243 (6)	−0.0095 (6)	−0.0045 (6)
O2	0.0311 (7)	0.0691 (10)	0.0259 (7)	−0.0202 (7)	−0.0042 (6)	−0.0020 (6)
O3	0.0475 (8)	0.0297 (7)	0.0593 (9)	−0.0152 (6)	−0.0261 (7)	−0.0046 (6)
N1	0.0272 (8)	0.0276 (8)	0.0286 (8)	−0.0128 (6)	−0.0113 (6)	0.0025 (6)
N2	0.0234 (7)	0.0236 (7)	0.0245 (7)	−0.0107 (6)	−0.0071 (6)	0.0021 (6)
N3	0.0278 (8)	0.0316 (8)	0.0358 (9)	−0.0140 (7)	−0.0134 (7)	0.0019 (6)
C1	0.0273 (9)	0.0232 (8)	0.0282 (9)	−0.0106 (7)	−0.0118 (7)	0.0031 (7)
C2	0.0408 (11)	0.0353 (10)	0.0262 (9)	−0.0162 (9)	−0.0121 (8)	0.0028 (8)
C3	0.0425 (11)	0.0388 (11)	0.0262 (10)	−0.0176 (9)	−0.0019 (8)	0.0022 (8)
C4	0.0298 (10)	0.0388 (11)	0.0366 (11)	−0.0174 (8)	−0.0023 (8)	0.0050 (8)
C5	0.0259 (9)	0.0302 (9)	0.0343 (10)	−0.0145 (8)	−0.0087 (7)	0.0044 (7)
C6	0.0233 (8)	0.0267 (9)	0.0234 (9)	−0.0116 (7)	−0.0066 (7)	0.0012 (7)
C7	0.0231 (8)	0.0228 (8)	0.0269 (9)	−0.0095 (7)	−0.0082 (7)	0.0016 (7)
C8	0.0314 (10)	0.0276 (9)	0.0356 (10)	−0.0109 (8)	−0.0084 (8)	0.0000 (8)
C9	0.0352 (11)	0.0488 (13)	0.0414 (12)	−0.0154 (10)	−0.0033 (9)	−0.0085 (9)

### Geometric parameters (Å, º)

**Table:**

S1—O1	1.4993 (13)	C2—C3	1.355 (3)
S1—C7	1.7965 (17)	C2—H2B	0.9300
S1—C8	1.8082 (18)	C3—C4	1.410 (3)
S2—O3	1.4255 (14)	C3—H3D	0.9300
S2—O2	1.4281 (14)	C4—C5	1.349 (3)
S2—N3	1.5954 (15)	C4—H4A	0.9300
S2—C6	1.7448 (17)	C5—H5A	0.9300
N1—C1	1.338 (2)	C6—C7	1.376 (2)
N1—C7	1.352 (2)	C8—C9	1.506 (3)
N2—C5	1.375 (2)	C8—H8A	0.9700
N2—C1	1.388 (2)	C8—H8B	0.9700
N2—C6	1.388 (2)	C9—H9A	0.9600
N3—H3A	0.83	C9—H9B	0.9600
N3—H3B	0.82	C9—H9C	0.9600
C1—C2	1.406 (2)		
			
O1—S1—C7	104.42 (8)	C5—C4—C3	121.08 (17)
O1—S1—C8	106.98 (8)	C5—C4—H4A	119.5
C7—S1—C8	97.74 (8)	C3—C4—H4A	119.5
O3—S2—O2	120.44 (9)	C4—C5—N2	118.26 (17)
O3—S2—N3	107.20 (8)	C4—C5—H5A	120.9
O2—S2—N3	108.79 (9)	N2—C5—H5A	120.9
O3—S2—C6	108.41 (8)	C7—C6—N2	104.92 (14)
O2—S2—C6	103.09 (8)	C7—C6—S2	130.38 (13)
N3—S2—C6	108.43 (8)	N2—C6—S2	124.68 (12)
C1—N1—C7	105.05 (14)	N1—C7—C6	112.36 (15)
C5—N2—C1	122.26 (14)	N1—C7—S1	118.80 (12)
C5—N2—C6	131.29 (15)	C6—C7—S1	128.78 (13)
C1—N2—C6	106.45 (13)	C9—C8—S1	110.33 (14)
S2—N3—H3A	112.6	C9—C8—H8A	109.6
S2—N3—H3B	112.2	S1—C8—H8A	109.6
H3A—N3—H3B	118.8	C9—C8—H8B	109.6
N1—C1—N2	111.22 (14)	S1—C8—H8B	109.6
N1—C1—C2	130.02 (16)	H8A—C8—H8B	108.1
N2—C1—C2	118.76 (15)	C8—C9—H9A	109.5
C3—C2—C1	118.91 (17)	C8—C9—H9B	109.5
C3—C2—H2B	120.5	H9A—C9—H9B	109.5
C1—C2—H2B	120.5	C8—C9—H9C	109.5
C2—C3—C4	120.71 (17)	H9A—C9—H9C	109.5
C2—C3—H3D	119.6	H9B—C9—H9C	109.5
C4—C3—H3D	119.6		
			
C7—N1—C1—N2	−0.09 (18)	O2—S2—C6—C7	−6.34 (19)
C7—N1—C1—C2	−179.63 (18)	N3—S2—C6—C7	−121.56 (17)
C5—N2—C1—N1	179.11 (15)	O3—S2—C6—N2	−59.80 (16)
C6—N2—C1—N1	−0.39 (18)	O2—S2—C6—N2	171.48 (14)
C5—N2—C1—C2	−1.3 (2)	N3—S2—C6—N2	56.26 (16)
C6—N2—C1—C2	179.21 (15)	C1—N1—C7—C6	0.56 (19)
N1—C1—C2—C3	179.69 (18)	C1—N1—C7—S1	177.83 (12)
N2—C1—C2—C3	0.2 (3)	N2—C6—C7—N1	−0.79 (19)
C1—C2—C3—C4	0.3 (3)	S2—C6—C7—N1	177.35 (13)
C2—C3—C4—C5	0.3 (3)	N2—C6—C7—S1	−177.72 (12)
C3—C4—C5—N2	−1.3 (3)	S2—C6—C7—S1	0.4 (3)
C1—N2—C5—C4	1.9 (3)	O1—S1—C7—N1	0.33 (15)
C6—N2—C5—C4	−178.78 (17)	C8—S1—C7—N1	110.16 (14)
C5—N2—C6—C7	−178.75 (16)	O1—S1—C7—C6	177.09 (16)
C1—N2—C6—C7	0.69 (17)	C8—S1—C7—C6	−73.08 (17)
C5—N2—C6—S2	3.0 (3)	O1—S1—C8—C9	−77.41 (15)
C1—N2—C6—S2	−177.60 (12)	C7—S1—C8—C9	174.87 (14)
O3—S2—C6—C7	122.38 (17)		

### Hydrogen-bond geometry (Å, º)

**Table:**

*D*—H···*A*	*D*—H	H···*A*	*D*···*A*	*D*—H···*A*
N3—H3*A*···O1^i^	0.83	2.07	2.888 (2)	171
N3—H3*B*···N1^ii^	0.82	2.24	3.026 (2)	161

Symmetry codes: (i) *x*−1, *y*, *z*; (ii) −*x*+1, −*y*+1, −*z*+1.
